# Patient with adrenal insufficiency due to a *de novo* mutation in the *NR0B1* gene

**DOI:** 10.1515/almed-2023-0018

**Published:** 2023-03-15

**Authors:** Daniel Bravo Nieto, Alba S. García Fernández, Noelia Díaz Troyano, Marina Giralt Arnaiz, Andrea Arias García, Paula Fernández Álvarez, Ariadna Campos Martorell, Roser Ferrer Costa, María Clemente León

**Affiliations:** Servicio de Bioquímica, Laboratorios Clínicos, Hospital Universitari Vall d’Hebron, Barcelona, Spain; Unidad de endocrinología pediátrica, Hospital Universitari Vall d’Hebron, Barcelona, Spain

**Keywords:** adrenal insufficiency, congenital adrenal hypoplasia, NR0B1

## Abstract

**Objectives:**

Congenital X-linked adrenal hypoplasia is a rare disease with a known genetic basis characterized by adrenal insufficiency, hypogonadotropic hypogonadism, and a wide variety of clinical manifestations.

**Case presentation:**

We present the case of a 26-day old male newborn with symptoms consistent with adrenal insufficiency, hyponatremia, and hyperkalemia. Following NaCl and fludrocortisone supplementation, the patient remained clinically stable. 17-OH-progesterone testing excluded congenital adrenal hyperplasia. The rest of hormones were within normal limits, except for adrenocorticotropic hormone (ACTH), which was significantly elevated, and aldosterone, which was below the reference value. Further testing included very long chain fatty acids to exclude adrenoleukodystrophy, the *CYP11B2* gene (aldosterone synthase), and an MRI to screen for other morphological abnormalities. All tests yielded normal results. Finally, after cortisol deficiency was detected, expanded genetic testing revealed a mutation in the *NR0B1* gene, which led to a diagnosis of congenital adrenal hypoplasia.

**Conclusions:**

Diagnosis of congenital adrenal hypoplasia is challenging due to the heterogeneity of both clinical manifestations and laboratory abnormalities. As a result, diagnosis requires close monitoring and genetic testing.

## Introduction

Congenital X-linked adrenal hypoplasia (X-linked AHC) (OMIM 300200) is a rare disease characterized by adrenal insufficiency (AI) in the early childhood and hypogonadotropic hypogonadism (HH), generally detected in puberty. Clinical manifestations are varied, with different levels of impairment of glucocorticoid, mineralocorticoid, and sex hormone metabolism.

Congenital X-linked AHC is caused by a mutation in the *NR0B1* gene (Xp21.2), which encodes the DAX1 protein, involved in signal transduction and expressed in the adrenal cortex, gonads, hypothalamus, and pituitary glands. Over 100 variants have been described, with different phenotypical effects, being AI, HH, and alterations in spermatogenesis the most frequent [1, 2]. Early genetic diagnosis of AHC may reduce morbimortality, warrants early hormone replacement, and enables the provision of genetic counseling to the family.

We report the case of a patient with symptoms of hypoaldosteronism. During follow-up, other abnormalities were detected, leading to a diagnosis of X-related AHC.

## Case presentation

A 26-day-old male newborn referred from other center with polyuric syndrome, dehydration, loss of 9% of birth weight, loss of appetite, and somnolence.

The newborn did not have a perinatal history of interest: second pregnancy, born by C-section at 39 weeks and 4 days, weight: 4,020 g (p95), size: 50.5 cm (p56), and normal neonatal screening results.

In-hospital laboratory analysis revealed Na^+^ 126 mmol/L (reference values: 136–146 mmol/L) and K^+^ 6.89 mmol/L (3.90–6.00 mmol/L). Fluid therapy was initiated with glucosaline at 5% and NaCl supplementation, resulting in clinical improvement. Hyponatremia exacerbated after switching from endovenous to oral therapy, and then endovenous therapy was reinitiated and fludrocortisone was added to the treatment. Hormone testing showed elevated levels of ACTH of 966 pg/mL (5–49 pg/mL), reduced levels of aldosterone, and 17-OH progesterone concentrations not suggestive of congenital adrenal hyperplasia (CAH).

At that point, the patient was referred to our center. Expanded hormone testing confirmed previous abnormalities. Cortisol concentrations were within the range of reference. Since ACTH and cortisol follow circadian rhythms, samples were collected in the early morning.

The analytical evolution of the patient is shown in Table 1.

**Table 1:** Analytical evolution of the patient.

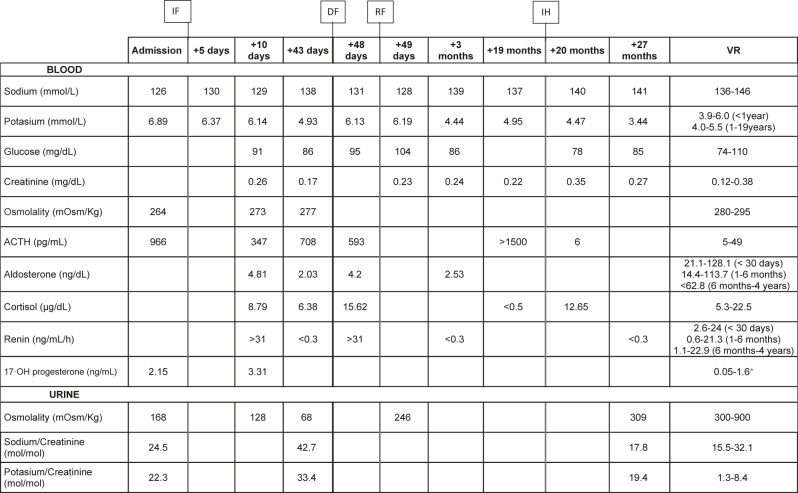

^a^An elevation of 17-OH progesterone above 10 ng/mL is suggestive of 21-hydroxylase–deficient congenital hyperplasia [10]. IF, initiation of fludrocortisone; DF, discontinuance of fludrocortisone; RF, reinitiation of fludrocortisone; IH, initiation of hydrocortisone; RV, reference values.

Patient’s course was favorable with fludrocortisone supplementation and endovenous NaCl (max. 2 mEq/kg at 1.2 cc/12 h), which was later switched to oral NaCl (3 mEq/kg). Polyuria persisted, with 4–6 cc/kg/h. A body weight gain of 350 g was achieved in 6 days. The patient was discharged with a regular follow-up plan.

At 6 weeks from the initial visit, sodium and potassium returned to normal levels. Fludrocortisone therapy was discontinued to eliminate drug interferences in hormone testing. The parents were instructed to seek emergency care if the clinical status of the newborn worsened. At that point, ACTH values were persistently elevated (708 pg/mL), although the result was obtained some days after it was decided to withdraw the treatment.

Five days after fludrocortisone was withdrawn, the parents reported that the child was more inactive and had lost appetite. Laboratory analysis revealed again hyponatremia (Na^+^ 131 mmol/L) and a weight loss of 300 g. Hormone testing showed once again elevated levels of ACTH (593 pg/mL), with cortisol being inappropriately normal for a patient with hyponatremia, low aldosterone, and elevated plasma renin (>31 ng/mL/h) (0.6–21.3 ng/mL/h).

On suspicion of primary adrenal insufficiency, and once CAH was excluded on the basis of normal 17-OH-progesterone concentrations, other potential congenital causes were considered. Very long chain fatty acid testing yielded normal results and excluded adrenoleukodystrophy. Size and morphologic abnormalities in the adrenal glands were excluded on MRI. On suspicion of a form of AI with isolated mineralocorticoid deficiency, a single variant test for the *CYP11B2* gene was carried out (informed consent was previously obtained). This gene encodes aldosterone synthase and has been associated with abnormalities in aldosterone activity [3, 4]. No pathogenic variants or variants of uncertain significance were identified in the encoding region of *CYP11B2* or other adjacent intronic regions. The patient remained clinically stable the following months with the fludrocortisone treatment.

Control laboratory analysis at 19 months and increased skin pigmentation led to the detection of cortisol deficiency (<0.5 μg/dL) (5.3–22.5 μg/dL). This finding led to a diagnostic suspicion of primary AI. Then, testing for genes associated with familial glucocorticoid deficiency was performed. The NGS panel was developed in the genetics laboratory of Hospital Universitari Vall d’Hebron, on the basis of the most prevalent abnormalities involving adrenal function. The panel includes the following genes: *CYP11A1*, *STAR*, *NR0B1*, *MC2R*, *MRAP*, *CYP17A1*, *MCM4*, *NNT*, and *TXNRD2.* Sequencing was performed on a MiSeq system (Illumina, Inc.). In *NR0B1*, a hemizygous cytosine to adenine mutation was detected at position 323 of exon 1 (c.323C>A p. (Ser108*)). This variant predicts an arginine-to-stop codon substitution at aminoacid 108 of the 4 X 67 AA tandem repeats region, and the generation of a truncated protein or loss of protein. Loss of function variants in *NR0B1* are a known pathogenic mechanisms in individuals with congenital X-linked AHC [1, 2].

This variant was not found in the databases searched: SNP, 1000 genomes, Exome Variant Server, Exome Aggregation Consortium, LOVD, and HGMD [5], [6], [7], [8], [9].

According to the criteria of the American College of Medical Genetics and the Genomics Association for Molecular Pathology for the interpretation and classification of variants, variant c.323C>A p. (Ser108*) is pathogenic.

The parents underwent genetic testing and none was a carrier. Therefore, the mutation was *de novo*.

After glucocorticoid deficiency was detected, hydrocortisone was added to the treatment and the patient remained clinically and analytically stable in subsequent follow-up visits, with a good weight and height development.

## Discussion

AI has different origins and clinical manifestations. It can originate at adrenal (primary AI), pituitary (secondary), or hypothalamic (tertiary) level. Partial forms may also occur, with isolated glucocorticoid, mineralocorticoid, or androgen deficiency. This heterogeneity makes the interpretation of biochemical and hormone test results challenging.

The clinical manifestations of AHC are varied, and glucocorticoid and mineralocorticoid deficiency may appear either simultaneously or consecutively, with different levels of abnormality. Initial symptoms are unspecific of CAH [10, 11]. Our patient presented with symptoms of hyponatremia without a medical history of interest. Neonatal screening was normal, although it does not include CAH in Catalonia. The most prevalent disease among newborns with weight loss, loss of appetite, and dehydration and with low levels of sodium and hyperkalemia is 21-hydroxylase–deficient CAH. In the case reported, normal 17-OH progesterone concentrations for postnatal age were inconsistent with this hypothesis [12].

ACTH elevation did not reach concentrations associated with glucocorticoid deficiency (generally >1,000 pg/mL) [13]; cortisol concentrations were within normal limits; external genitalia were normal; and only aldosterone was low. These findings led to an initial diagnostic suspicion of isolated mineralocorticoid deficiency.

The course of the patient demonstrates that manifestations of hormone deficiency may appear progressively. In this case, mineralocorticoid deficiency was the first manifestation, with the patient having shown symptoms from 3 weeks of life, whereas glucocorticoid deficiency was not detected until 19 months of life. In these cases, ACTH elevation may precede the detection of glucocorticoid deficiency, thereby suggesting the presence of AHC. At clinical level, mucosal pigmentation may support suspicion as it indicates chronically elevated levels of ACTH. ACTH is originated from proopiomelanocortin, a prohormone that converts into ACTH and melanotropin [1].

The elevation of plasma renin activity in the case described is interpreted as a compensatory response to aldosterone deficiency, when low blood pressure is not corrected. This elevation disappears (with values reaching undetectable levels) following the administration of fludrocortisone. Aldosterone secretion is primarily regulated by the renin–angiotensin–aldosterone system, although it is also modulated by ACTH, albeit with a lower intensity.

## Lessons learned

– On suspicion of an abnormal adrenal function, when symptoms and initial testing are inconclusive, analytical and clinical follow-up is essential for diagnosis.
– In these cases, elevated levels of ACTH and plasma renin, along with normal or low levels of cortisol and aldosterone, could be suggestive of AI. Androgens and 17-OH progesterone concentrations would guide diagnosis to AHC.
– As in previous cases, progressive presentation of symptoms makes diagnosis difficult and genetic testing is required for a final diagnosis. Then, it is important to identify the carrier and provide genetic counseling.
